# Risk Factors Associated With COVID-19 Outcomes Among People With Intellectual and Developmental Disabilities Receiving Residential Services

**DOI:** 10.1001/jamanetworkopen.2021.12862

**Published:** 2021-06-08

**Authors:** Scott D. Landes, Margaret A. Turk, Marco R. Damiani, Philip Proctor, Sarah Baier

**Affiliations:** 1 Department of Sociology and Aging Studies Institute, Maxwell School of Citizenship and Public Affairs, Syracuse University, Syracuse, New York; 2 Department of Physical Medicine & Rehabilitation, SUNY Upstate Medical University, Syracuse, New York; 3 AHRC New York City, New York, New York

## Abstract

**Importance:**

Although there is evidence of more severe COVID-19 outcomes, there is no information describing the risk factors for COVID-19 diagnosis and/or mortality among people with intellectual and developmental disabilities (IDD) receiving residential support services in the US.

**Objective:**

To identify associations between demographic characteristics, residential characteristics, and/or preexisting health conditions and COVID-19 diagnosis and mortality for people with IDD receiving residential support services.

**Design, Setting, and Participants:**

This cohort study tracked COVID-19 outcomes for 543 individuals with IDD. Participants were receiving support services from a single organization providing residential services in the 5 boroughs of New York City from March 1 to October 1, 2020. Statistical analysis was performed from December 2020 to February 2021.

**Exposures:**

Resident-level characteristics, including age, sex, race/ethnicity, disability status, residential characteristics, and preexisting medical conditions.

**Main Outcomes and Measures:**

COVID-19 diagnosis was confirmed by laboratory test. COVID-19 mortality indicated that the individual died from COVID-19 during the course of the study. Logistic regression models were used to evaluate associations between demographic characteristics, residential characteristics, and preexisting health conditions and COVID-19 diagnosis and mortality.

**Results:**

Among the 543 individuals with IDD in the study, the median (interquartile range) age was 57.0 (45-65) years; 217 (40.0%) were female, and 274 (50.5%) were Black, Asian/Pacific Islander, American Indian or Alaskan Native, or Hispanic. The case rate was 16 759 (95% CI, 13 853-20 131) per 100 000; the mortality rate was 6446 (95% CI, 4671-8832) per 100 000; and the case-fatality rate was 38.5% (95% CI, 29.1%-48.7%). Increased age (odds ratio [OR], 1.04; 95% CI, 1.02-1.06), Down syndrome (OR, 2.91; 95% CI, 1.49-5.69), an increased number of residents (OR, 1.07; 95% CI, 1.00-1.14), and chronic kidney disease (OR, 4.17; 95% CI, 1.90-9.15) were associated with COVID-19 diagnosis. Heart disease (OR, 10.60; 95% CI, 2.68-41.90) was associated with COVID-19 mortality.

**Conclusions and Relevance:**

This study found that, similar to the general population, increased age and preexisting health conditions were associated with COVID-19 outcomes for people with IDD receiving residential support services in New York City. As with older adults living in nursing homes, number of residents was also associated with more severe COVID-19 outcomes. Unique to people with IDD was an increased risk of COVID-19 diagnosis for people with Down syndrome.

## Key Points

**Question:**

What individual and residential characteristics are associated with COVID-19 outcomes for people with intellectual and developmental disabilities receiving residential services?

**Findings:**

In this cohort study of 543 adults with intellectual and developmental disabilities receiving residential services in New York City, age, larger residential settings, Down syndrome, and chronic kidney disease were associated with COVID-19 diagnosis. Heart disease was associated with COVID-19 mortality.

**Meaning:**

This study’s findings suggest that risk factors for COVID-19 diagnosis and mortality for people with intellectual and developmental disabilities receiving residential services are similar to (age, preexisting conditions, size of residence) and unique from (Down syndrome) those reported in the general population.

## Teaser

This cohort study examines risk factors associated with COVID-19 positivity and mortality rates among individuals with intellectual and developmental disabilities receiving residential support services in New York City.

## Introduction

There is mounting empirical evidence that people with intellectual and developmental disabilities (IDD) in the US, a vulnerable health population,^1^ are experiencing more severe COVID-19 outcomes than the general population. An early study using real-time electronic medical record data for individuals diagnosed with COVID-19 reported that people with IDD had higher prevalence of comorbidities (respiratory diseases; endocrine, nutritional, and metabolic diseases; and circulatory diseases) associated with more severe COVID-19 outcomes, and had a higher case-fatality rate at ages 0 to 17 years and 18 to 74 years.^2^ A second study using data for New York State reported that the COVID-19 case rate was 4.1 times higher and case-fatality rate 1.9 times higher for people with IDD living in residential group homes than for the state overall.^3^ A more recent study used data from California and reported that the COVID-19 outcomes for people with IDD varied by residential setting, with higher case rates in settings with more residents and higher case-fatality rates in settings providing skilled nursing care.^4^

Although critical evidence, data used in these early studies either did not include demographic characteristics and preexisting conditions^3^ or did not provide these data in a way that permitted examination of whether these factors were associated with COVID-19 outcomes among people with IDD.^2,4^ Studies on the general population consistently report more severe COVID-19 outcomes among older adults^5,6,7,8^ as well as those with preexisting medical conditions.^9^ There is also some evidence of disproportionate effects of COVID-19 on Black and Hispanic people in the US.^8,10,11,12,13^ It is reasonable to think that these same demographic and health factor associations are present among those with IDD. In addition, it is possible that similar to older adults living in nursing homes, people with IDD receiving direct, difficult-to-physically distance support in certain congregate settings,^14,15,16^ especially those with a greater number of residents,^17,18^ may be at greater risk during the pandemic.^4,19^ Unique to people with IDD, one study from the UK identified people with Down syndrome, a particular type of IDD, may be at increased risk of COVID-19 death.^20^

To better understand COVID-19 outcomes among people with IDD, the present study used a sample of adults with IDD receiving residential support services in New York City, the epicenter of the pandemic in the US. Based upon the results of prior studies, we hypothesized that more severe COVID-19 outcomes would be associated with increased age, residential characteristics, Down syndrome, and preexisting health conditions.

## Methods

### Data

Data for this study are from AHRC New York City (AHRCNYC). AHRCNYC is a nonprofit organization with 5000 staff members that provided ongoing long-term support services to 8275 individuals across the 5 boroughs of New York City during the course of the study. Of those receiving support services from AHRCNYC, 554 people received residential services at the beginning of the pandemic in 3 distinct types of settings. Supportive settings provided up to 23 hours per week of staff support, with a mean of 8 to 10 hours per week, for assistance with activities of independent daily living. Supervised settings provided 24/7 on-site staff support for assistance with activities of daily living and may include intermittent, but not 24-hour, nursing care. Intermediate care facilities (ICF) provided 24/7 onsite staff support for complex medical needs that required on-site 24-hour nursing coverage.

AHRCNYC tracked COVID-19 outcomes from the beginning of the pandemic through the current period. For this study, we used fully deidentified AHRCNYC COVID-19 Response Data from March 1 through October 1, 2020 on 543 people with IDD who received residential supports for the duration of the study. Eleven individuals were excluded as they transitioned to living with family members during the course of the study. AHRCNYC residential and nursing staff reviewed the case files for each resident to determine demographic and residential characteristics, preexisting conditions, and COVID-19 status. Data quality checks were performed by the Directors of Residential Nursing and Enterprise Technology Strategy to ensure the accuracy of the data. The study was reviewed by the Syracuse University institutional review board and determined to be exempt, including waiver of informed consent, as it used fully deidentified data. This study followed the Strengthening the Reporting of Observational Studies in Epidemiology (STROBE) reporting guideline for cohort studies.

### Measures

COVID-19 diagnosis indicated the individual had been diagnosed with COVID-19 via laboratory test during the course of the study. Screening for COVID-19 positive status among AHRCNYC residents involved symptomatic testing without contact tracing. Thus, results do not indicate incidence. COVID-19 mortality indicated that the individual died from COVID-19 during the course of the study.

Age was coded in single years. Sex was coded 1 for female and 0 for male. As there has been some evidence of more severe COVID-19 outcomes among Black and Hispanic individuals in the US, we included a measure for race/ethnicity with data originating from the case file review. Because there was a small number of events among the minority racial-ethnic groups (Black, Asian/Pacific Islander, American Indian or Alaskan Native, or Hispanic) included in the study, race/ethnicity was coded dichotomously as 1 for racial-ethnic minority status and 0 for non-Hispanic White. Type of residence included Supportive, Supervised, and ICF. Number of residents was coded continuously.

Although all individuals in the study had a *Diagnostic and Statistical Manual of Mental Disorders* (Fifth Edition) (*DSM-5*) diagnosis of IDD, 2 separate measures differentiate whether people did (coded 1) or did not (coded 0) also have Down syndrome or cerebral palsy.

AHRCNYC collected data on preexisting health conditions the Centers for Disease Control and Prevention (CDC) reported at the time of the study as having the “strongest and most consistent evidence” of more severe COVID-19 outcomes in the general population: cancer,^21,22^ chronic kidney disease,^23,24^ chronic obstructive pulmonary disease (COPD),^25,26^ heart conditions,^26,27^ obesity (indicated by body mass index, calculated as weight in kilograms divided by height in meters squared, greater than 30),^28,29^ pregnancy,^30,31^ sickle cell disease,^32,33^ immunocompromised from solid organ transplant,^34,35^ type 2 diabetes,^23,36^ or current smoking.^27,37^ Two of these conditions were excluded as no individuals with pregnancy or sickle cell disease were COVID-19 positive during the course of the study. Each of the remaining 8 preexisting conditions were coded dichotomously indicating the individual either did (coded 1) or did not (coded 0) have the condition.

### Statistical Analysis

All analysis examines COVID-19 outcomes among people with IDD receiving residential services from AHRCNYC. Initial analysis focused on the distribution of all measures for the entire sample, as well as for people who were or were not diagnosed with COVID-19. In addition to basic distributions, we calculated the case rate ([cases/sample size] × 100 000), mortality rate ([deaths/sample size] × 100 000), and case-fatality rate (deaths/cases) for the sample. The 95% CIs for the IDD sample were calculated using the Wilson method because of the small sample size. As a point of comparison, these rates were juxtaposed with those of New York City for the same period. Data for New York City was from the Johns Hopkins Center for Systems Science and Engineering COVID-19 data.^38^ The population of New York City was based upon US Census 2019 estimates.^39^

Analysis of COVID-19 diagnosis status included the entire sample. Analysis of COVID-19 mortality included only those individuals who were diagnosed with COVID-19 during the course of the study. Univariable and multivariable analyses of both outcomes were conducted with logistic regression. A Firth logistic regression model, with penalized likelihood estimation method, was specified for analysis of COVID-19 mortality as one risk factor was a quasicomplete separator.^40^

Owing to the small number of events for both outcomes, we used a purposeful selection strategy to create as parsimonious a model as possible for multivariable analysis, ensuring at least 5 events per independent variable^41^ (models only included measures that had a sufficient number of events to ensure reasonable standard errors [5 events]), and a *P* value < .25.^22,42,43^ For analysis of COVID-19 mortality, type of residence was collapsed into a dichotomous measure indicating ICF (coded 1) and supportive or supervised (coded 0) to ensure a sufficient number of events. The measure for immunocompromised from solid organ transplant was not included in analysis of COVID-19 mortality as no individuals with this condition died during the course of the study. *P* values were 2-sided, and a significance level was set at *P* < .05. All analysis was conducted with Stata statistical software version 16.0 (StataCorp) from December 2020 to February 2021.

## Results

Among the 543 individuals with IDD in the study, the median (interquartile range) age was 57.0 (45-65) years; 217 (40.0%) were female, and 274 (50.5%) were Black, Asian/Pacific Islander, American Indian or Alaskan Native, or Hispanic. The distribution of all study variables is reported in Table 1.

**Table 1.** Distribution of Demographic and Health Characteristics^a^

Variable	No. (%)		
	Entire sample (N = 543)	COVID-19 positive (n = 91)	Not COVID-19 positive (n = 452)
Age, median (IQR), y	57 (45-65)	64 (56-69)	55 (44-64)
Sex			
Female	217 (40.0)	39 (42.9)	178 (39.4)
Male	326 (60.0)	52 (57.1)	274 (60.6)
Race/ethnicity			
Non-Hispanic White	269 (49.5)	55 (60.4)	214 (47.4)
Black, Asian/Pacific Islander, American Indian or Alaskan Native, or Hispanic	274 (50.5)	36 (39.6)	238 (52.7)
Type of residence			
Supportive	107 (19.7)	11 (12.1)	96 (21.2)
Supervised	376 (69.2)	61 (67.0)	315 (69.7)
Intermediate care facilities	60 (11.1)	19 (20.9)	41 (9.1)
No. of residents, mean (SE) [range]	7.21 (0.19) [1-19]	8.36 (0.47) [1-19]	6.97 (0.21) [1-19]
Down syndrome	56 (10.3)	20 (22.0)	36 (8.0)
Cerebral palsy	50 (9.2)	8 (8.8)	42 (9.3)
Preexisting conditions			
Cancer	37 (6.8)	10 (11.0)	27 (6.0)
Chronic kidney disease	36 (6.6)	17 (18.7)	19 (4.2)
COPD	26 (4.8)	10 (11.0)	16 (3.5)
Heart disease	95 (17.5)	20 (22.0)	75 (16.6)
Immunocompromised^b^	15 (2.7)	2 (2.2)	13 (2.9)
Obesity^c^	158 (29.1)	22 (24.2)	136 (30.1)
Currently smoking	32 (5.9)	2 (2.2)	30 (6.6)
Type 2 diabetes	103 (19.0)	17 (18.7)	86 (19.0)

Abbreviations: COPD, chronic obstructive pulmonary disease; BMI, body mass index (calculated as weight in kilograms divided by height in meters squared); IQR, interquartile range.

^a^ Table data based on AHRCNYC Residential Services COVID-19 data.

^b^ Immunocompromised due to solid organ transplant.

^c^ Obesity was defined as having a BMI greater than 30.

During the course of the study, 91 individuals (16.8%) were diagnosed with COVID-19, and 35 individuals (6.4%) who were diagnosed with COVID-19 died from COVID-19. Thus, the case rate was 16 759 (95% CI, 13 853-20 131) per 100 000; the mortality rate was 6446 (95% CI, 4671-8832) per 100 000; and the case-fatality rate was 38.5% (95% CI, 29.1%-48.7%). As a point of comparison, for New York City overall for the same time period, the case rate was 2978 (95% CI, 2956-2979) per 100 000; the mortality rate was 286 (95% CI, 282-289) per 100 000; and the case-fatality rate was 9.6% (95% CI, 9.5%-9.7%).

Distribution by type of residence was 107 individuals (19.7%) living in a supportive residence, 376 individuals (69.2%) living in a supervised setting, and 60 individuals (11.1%) living in an ICF. The mean number of residents was 7.21 (SE, 0.19; range, 1-19). Although all individuals in the sample had a *DSM-5* diagnosis of IDD, 56 (10.3%) also had Down syndrome, and 50 (9.2%) also had cerebral palsy. The most prevalent preexisting conditions were obesity (158 individuals [29.1%]), type 2 diabetes (103 individuals [19.0%]), and heart disease (95 individuals [17.5%]). Less prevalent preexisting medical conditions included cancer (37 individuals [6.8%]); chronic kidney disease (36 individuals [6.6%]); COPD (26 individuals [4.8%]), and immunocompromised due to organ transplant (15 individuals [2.7%]). Individuals currently smoking represented 5.9% (32 individuals) of the sample (Table 1).

### COVID-19 Positive Status

Table 1 also includes distributions for all study measures by COVID-19 diagnosis status. Compared with those not diagnosed with COVID-19, those who were COVID-19 positive were older (median [interquartile range] age: 61 [56-69] years for individuals who were COVID-19 positive vs 55 (44-64) years for individuals who were not COVID-19 positive); included a higher percentage of those who were non-Hispanic White (55 of 91 individuals [60.4%] who were COVID-19 positive vs 214 of 452 individuals [47.4%] who were not positive), lived in ICFs (19 of 91 individuals [20.9%] who were positive vs 41 of 452 individuals [9.1%] who were not positive), or had Down syndrome (20 of 91 individuals [22.0%] who were positive vs 36 of 452 individuals [8.0%] who were not positive); lived in settings with more residents (mean [SE] number of residents: 8.36 [0.47] residents for individuals who were positive vs 6.97 [0.21] for individuals who were not); and had a higher prevalence of cancer (10 of 91 individuals [11.0%] who were positive vs 27 of 452 individuals [6.0%] who were not positive), chronic kidney disease (17 of 91 individuals [18.7%] who were positive vs 19 of 452 individuals [4.2%] who were not positive), COPD (10 of 91 individuals [11.0%] who were positive vs 16 of 452 individuals [3.5%] who were not positive), or heart disease (20 of 91 individuals [22.0%] who were positive vs 75 of 452 individuals [16.6%] who were not positive).

Univariable and multivariable results for COVID-19 diagnosis are reported in Table 2. In univariable analysis, increased risk of COVID-19 diagnosis was associated with increased age (odds ratio [OR], 1.04; 95% CI, 1.03-1.06; *P* < .001), living in an ICF as opposed to a supportive or supervised setting (supportive vs ICF: OR, 0.25; 95% CI, 0.11-0.57; *P* = .001; supervised vs ICF: OR, 0.42; 95% CI, 0.23-0.77; *P* = .005), an increased number of residents (OR, 1.07; 95% CI, 1.02-1.12; *P* = .006), Down syndrome (OR, 3.26; 95% CI, 1.78-5.94; *P* < .001), chronic kidney disease (OR, 5.24; 95% CI, 2.60-10.53; *P* < .001), and COPD (OR, 3.36; 95% CI, 1.47-7.68; *P* = .004). Individuals who were Black, Asian/Pacific Islander, American Indian or Alaskan Native, or Hispanic had comparatively less risk of COVID-19 diagnosis (OR, 0.59; 95% CI, 0.37-0.93; *P* = .02) than non-Hispanic White individuals. In multivariable analysis, the risk of COVID-19 diagnosis increased by 4% with each year increase with age (OR, 1.04; 95% CI, 1.02-1.06; *P* < .001), increased by 7% with each increase in number of residents (OR, 1.07; 95% CI, 1.00-1.14; *P* = .04), was 2.9 times higher for people with than without Down syndrome (OR, 2.91; 95% CI, 1.49-5.69; *P* = .002), was 4.2 times higher for people with than without chronic kidney disease (OR, 4.17; 95% CI, 1.90-9.15; *P* < .001), but was 53% less likely in a supervised setting than in an ICF (OR, 0.48; 95% CI, 0.24-0.94; *P* = .03).

**Table 2.** Factors Associated With COVID-19 Positive Status^a^

Variable	Univariable		Multivariable	
	OR (95% CI)	*P* value	OR (95% CI)	*P* value
Age	1.04 (1.03-1.06)	<.001	1.04 (1.02-1.06)	<.001
Sex				
Male	[Reference]	NA	NA	NA
Female	1.15 (0.73-1.82)	.44	NA	NA
Black, Asian/Pacific Islander, American Indian or Alaskan Native, or Hispanic	0.59 (0.37-0.93)	.02	0.73 (0.43-1.24)	.25
Type of residence				
ICF	[Reference]	NA	[Reference]	NA
Supportive	0.25 (0.11-0.57)	.001	0.53 (0.20-1.42)	.21
Supervised	0.42 (0.23-0.77)	.005	0.48 (0.24-0.94)	.03
No. of residents	1.07 (1.02-1.12)	.006	1.07 (1.00-1.14)	.04
Down syndrome	3.26 (1.78-5.94)	<.001	2.91 (1.49-5.69)	.002
Cerebral palsy	0.94 (0.43-2.08)	.88	NA	NA
Preexisting conditions				
Cancer	1.94 (0.91-4.17)	.09	0.58 (0.22-1.55)	.28
Chronic kidney disease	5.24 (2.60-10.53)	<.001	4.17 (1.90-9.15)	<.001
COPD	3.36 (1.47-7.68)	.004	2.32 (0.84-6.38)	.10
Heart disease	1.42 (0.81-2.47)	.22	0.68 (0.34-1.37)	.28
Immunocompromised^b^	0.76 (0.17-3.42)	.74	NA	NA
Obesity^c^	0.74 (0.44-1.25)	.26	NA	NA
Currently smoking	0.32 (0.07-1.35)	.12	NA	NA
Type 2 diabetes	0.98 (0.55-1.74)	.94	NA	NA

Abbreviations: BMI, body mass index (calculated as weight in kilograms divided by height in meters squared); COPD, chronic obstructive pulmonary disease; ICF, intermediate care facility; NA, not applicable.

^a^ Table data based on AHRCNYC Residential Services COVID-19 data.

^b^ Immunocompromised due to solid organ transplant.

^c^ Obesity was defined as having a BMI greater than 30.

Table 3 reports supplementary analysis of the multivariable model with age as categorical to clarify age trends. Compared with those aged 19 to 39 years, the risk of COVID-19 diagnosis was 3.6 times higher (95% CI, 1.5-9.0) among those aged 60 to 69 years, and 3.2 times higher (95% CI, 1.2-8.6) among those aged 70 years and older .

**Table 3.** Factors Associated With COVID-19 Positive Status^a^

Variable	Multivariable	
	OR (95% CI)	*P* value
Age, y		
19-39	1 [Reference]	NA
40-49	0.93 (0.27-3.19)	.91
50-59	2.02 (0.80-5.10)	.14
60-69	3.63 (1.47-8.97)	.005
≥70	3.22 (1.20-8.60)	.02
Black, Asian/Pacific Islander, American Indian or Alaskan Native, or Hispanic	0.71 (0.42-1.20)	.21
Type of residence		
ICF	1 [Reference]	NA
Supportive	0.49 (0.18-1.32)	.16
Supervised	0.45 (0.23-0.89)	.02
No. of residents	1.07 (1.00-1.14)	.045
Down syndrome	2.57 (0.22-5.08)	.007
Preexisting conditions		
Cancer	0.59 (0.91-1.58)	.30
Chronic kidney disease	4.17 (1.89-9.22)	<.001
COPD	2.77 (1.00-7.68)	.05
Heart disease	0.72 (0.36-1.45)	.36

Abbreviations: COPD, chronic obstructive pulmonary disease; ICF, intermediate care facility; NA, not applicable.

^a^ Table data based on AHRCNYC Residential Services COVID-19 data.

### COVID-19 Deaths

Univariable and multivariable results for factors associated with COVID-19 mortality among the 91 cases of individuals diagnosed with COVID-19 are reported in Table 4. There were 2 factors associated with COVID-19 mortality in univariable analysis. Risk of COVID-19 morality was 4.4 times higher (95% CI, 1.1-18.4) among those with COPD, and 16.7 times higher (95% CI, 4.4-63.6) among those with heart disease . In the multivariable model, only heart disease was associated with increased COVID-19 mortality, with a 10.6 increased risk (95% CI, 2.7-41.9). Follow-up analysis supported the association between heart disease and COVID-19 mortality. Heart disease accounted for 17 out of 35 (48.6%) of all COVID-19 deaths. In addition, 17 out of 20 (85%) of those with heart disease who were diagnosed with COVID-19 died during the course of the study.

**Table 4.** Factors Associated With COVID-19 Death^a^

Variable	Univariable, OR (95% CI)	*P* value	Multivariable, OR (95% CI)	*P* value
Age	1.03 (1.00-1.06)	.09	0.99 (0.95-1.03)	.67
Sex				
Male	[Reference]	NA	NA	NA
Female	1.00 (0.43-2.35)	>.99	NA	NA
Black, Asian/Pacific Islander, American Indian or Alaskan Native, or Hispanic	0.57 (0.23-1.38)	.21	0.86 (0.31-2.35)	.77
ICF	1.59 (0.57-4.42)	.37	NA	NA
No. of residents	1.08 (0.98-1.19)	.12	1.09 (0.97-1.21)	.15
Down syndrome	2.39 (0.87-6.56)	.09	3.35 (0.74-7.50)	.15
Cerebral palsy	1.68 (0.39-7.19)	.49	NA	NA
Preexisting conditions				
Cancer	4.42 (1.06-18.42)	.04	2.29 (0.46-11.41)	.31
Chronic kidney disease	1.15 (0.39-3.37)	.80		
COPD	4.42 (1.06-18.42)	.04	1.52 (0.23-10.28)	.67
Heart disease	16.69 (4.37-63.64)	<.001	10.60 (2.68-41.90)	.001
Obesity^b^	1.47 (0.55-3.88)	.44	NA	NA
Currently smoking	1.62 (0.10-26.72)	.74	NA	NA
Type 2 diabetes	1.55 (0.53-4.48)	.42	NA	NA

Abbreviations: BMI, body mass index (calculated as weight in kilograms divided by height in meters squared); COPD, chronic obstructive pulmonary disease; ICF, intermediate care facility; NA, not available.

^a^ Table data based on AHRCNYC Residential Services COVID-19 data.

^b^ Obesity was defined as having a BMI greater than 30.

## Discussion

To our knowledge, this study provided the first examination into the risk factors associated with COVID-19 outcomes among people with IDD receiving residential support services in New York City. The fact that the case rate, case-fatality rate, and mortality rate were substantially higher for people with IDD living in residential settings than for New York City overall is consistent with results from prior studies documenting increased COVID-19 risk for people with IDD and emphasizes the urgent need to prioritize vaccination allocation for this population.^44,45^

Analysis of risk factors associated with COVID-19 among people with IDD found similarities to and differences from the general population. These results provide critical information needed to ensure the health and safety of people with IDD during the pandemic. First, it is important to recognize that as with the general population, increased age and preexisting conditions are associated with more severe COVID-19 outcomes for people with IDD. This study pinpoints chronic kidney disease and heart disease as more strongly associated with COVID-19 diagnosis and death, respectively. For people with IDD, both chronic kidney disease and heart disease are reported to have similar overall prevalence compared with the general population.^46,47^ However, people with IDD may be at greater risk for advanced chronic kidney disease,^48^ and specific types of heart disease may be more common in older adults with IDD, such as heart failure.^49^ As with other reports of comorbidities, details about severity or specific diagnosis were not collected. Thus, we are unable to determine if associations with COVID-19 diagnosis and mortality are related to specifics within the broad categories. Preexisting conditions such as cancer and COPD also were associated with COVID-19 diagnosis in univariable analysis, although these conditions are typically less prevalent in adults with IDD.^49^ Although these associations were eliminated in multivariable models controlling for age and other comorbidities, it is important to recognize these associations exist and ensure that people with IDD who have any conditions associated with increased COVID-19 risk are receiving appropriate medical care during the pandemic.^50,51^

A second result deserving attention is the increased risk of COVID-19 severity for people with Down syndrome. In multivariable models, people with Down syndrome in this study were 2.9 times more likely to be diagnosed with COVID-19 and 3.4 times more likely to die from COVID-19. Although the association between Down syndrome and mortality was not statistically significant. Combined with the evidence from an earlier study from the UK,^20^ these results indicate an immediate need for all parties involved in the care of people with Down syndrome to take extra precautions to ensure the health and safety of this population.

The final result needing emphasis was the association between number of residents living in a setting and COVID-19 diagnosis. Based upon this result, it is important to note that size of residence, per se, is an essential consideration for agencies supporting people with IDD in congregate living settings. It is possible this outcome simply reflects that, as with older adults living in nursing homes,^18^ people with IDD living in settings with more residents necessarily experience a higher level of interaction with others, both other residents and support staff.^4^ However, consideration of community-level factors,^17^ and a more granular assessment of the risk profiles (eg, personal support needs, nursing and/or medical needs) of people living in settings of all sizes is essential in fully understanding whether the number of residents alone is an actual risk for more severe outcomes, or possibly a proxy for other factors. Although type of residential service was associated with COVID-19 diagnosis in univariable analysis, this association was either eliminated (supportive vs ICF) or severely attenuated (supervised vs ICF) in multivariable analysis.

### Limitations

This study has some limitations. Similar to other studies exploring COVID-19 outcomes among people with IDD, the primary limitation derives from the paucity of available data on this population.^52^ To help fill this gap, we relied on a sample of adults with IDD receiving residential services. Although employing a strategy to ensure parsimonious multivariable modeling, the infrequent number of events likely influenced results, especially for analysis of COVID-19 mortality. Thus, out of an abundance of caution, we feel it important to recognize the factors associated with COVID-19 outcomes that were statistically significant in either univariable or multivariable models. Although the small number of events resulted in wide CIs for the association between heart disease and COVID-19 mortality, it is important to recognize that this was a quasicomplete risk factor, with 85% of those with heart disease who had a COVID-19 diagnosis dying during the course of the study. Thus, we are confident in identifying heart disease as a risk factor for COVID-19 mortality in this study. A second limitation is that COVID-19 positive status is based upon symptomatic testing without contact tracing. It is probable that some AHRCNYC residents were asymptomatic positive during the period of the study, meaning that the reported diagnosis rate is not a true incidence rate, and as with much of the current COVID-19 data, is likely an underestimate. A third limitation of note is the geographic locale of the sample. All individuals in the study resided in the 5 boroughs of New York City. Although the passing of time is showing that the effect of the pandemic within New York City is not as unique as was first thought, we cannot generalize results of the study beyond this locale, or for that matter, beyond the AHRCNYC. A final limitation is that the measure used for body mass index in the study, while recommended by the CDC and indicative of more severe COVID-19 outcomes in the general population, may not accurately measure obesity among people with IDD.^53^

## Conclusions

The substantially higher case rate and case-fatality rate reported for people with IDD in this study further emphasizes the necessity to prioritize this population for COVID-19 vaccine allocation. In addition, results underscore that COVID-19 diagnosis should prompt close monitoring and consideration of hospitalization if respiratory symptoms develop for all people with IDD, but especially for those who are older, have preexisting medical conditions and/or Down syndrome, or live in settings with more residents. Standard preventive measures must be employed. But the additional risks reported in this study suggest that it may be necessary to adapt the unique basic and complex daily living care and support needs that exist in community-based congregate living arrangements for people with IDD in ways that minimize infection risk of residents and staff.
